# Coronary angiography identification of circumflex artery bleeding mimicking left ventricular rupture after mitral valve repair: a case report

**DOI:** 10.1186/s13019-026-04535-6

**Published:** 2026-07-15

**Authors:** Kohei Furusawa, Teruyoshi Kume, Shiro Uemura, Atsushi Tabuchi, Yoshiko Watanabe, Takahiko Yamasawa, Michihiro Okuyama, Noriaki Kuwada, Taishi Tamura, Samuel Jung, Daiki Hashiguchi, Susumu Doita, Sohei Hamanaka, Dai Une

**Affiliations:** 1 Department of Cardiovascular Surgery, Kawasaki Medical School Hospital, 577, Matsushima, Kurashiki- shi, Okayama-ken, 701-0192 Japan; 2 Department of Cardiology, Kawasaki Medical School Hospital, 577, Matsushima, Kurashiki- shi, Okayama-ken, 701-0192 Japan

**Keywords:** Mitral valve surgery, Circumflex artery injury, Left ventricular rupture, Postoperative bleeding, Coil embolization, Coronary angiography

## Abstract

**Background:**

Left ventricular rupture after mitral valve surgery is a rare but catastrophic complication with an extremely high mortality rate. Cases developing massive postoperative bleeding often require urgent surgical re-exploration. However, other causes of periannular bleeding, including iatrogenic coronary artery injury, should also be considered. Herein, we describe the case of a patient with periannular bleeding after mitral valve repair initially suspected to be left ventricular rupture but ultimately diagnosed as a distal left circumflex artery injury and successfully treated with coil embolization.

**Case presentation:**

A 79-year-old male underwent mitral valve repair, pulmonary vein isolation, and left atrial appendage closure for severe mitral regurgitation and paroxysmal atrial fibrillation. During surgery, tissue fragility was noted and a subepicardial hematoma was observed around the circumflex artery, although no active bleeding was detected. Massive drainage bleeding occurred approximately 30 min after surgery. Left ventricular rupture and myocardial injury related to cardiac manipulation were suspected. Contrast-enhanced computed tomography revealed contrast leakage around the mitral annulus; however, the source of the bleeding could not be identified. Coronary angiography revealed contrast extravasation from the distal left circumflex artery. Selective coronary coil embolization resulted in an immediate reduction in bleeding. The patient subsequently underwent re-exploration surgery to remove the intrathoracic hematoma without additional hemostatic intervention. The postoperative course was uneventful, and the patient was discharged on postoperative day 18.

**Conclusions:**

Coronary artery injury should be considered in the differential diagnosis of cases of suspected left ventricular rupture after mitral valve surgery. When hemodynamic conditions allow, coronary angiography may be useful for identifying the bleeding source and avoiding unnecessary surgical re-exploration.

## Background

Left ventricular rupture following mitral valve surgery is a rare but life-threatening complication, with reported incidence and mortality rates of 0.5–2% and 65–75%, respectively [1, 2]. A large series of 2560 patients undergoing mitral valve replacement reported an incidence rate of 0.8%, with a mortality rate as high as 86% [3]. Given this high mortality, prompt diagnosis and emergency surgical intervention are usually required when massive postoperative bleeding occurs.

However, periannular bleeding after mitral valve surgery may also result from injury to adjacent anatomical structures. The left circumflex artery (LCx) runs along the atrioventricular groove, in close proximity to the posterior mitral annulus. Anatomical studies have demonstrated that the distance between the LCx and mitral annulus can be extremely short, occasionally as little as 1 mm, making the artery vulnerable to injury during mitral valve surgery [4].

Several case series and reviews have reported iatrogenic injury of the LCx during mitral valve surgery [4, 5]. The proposed mechanisms include annular sutures, compression by prosthetic rings, traction during cardiac manipulation, or coronary artery distortion.

We report a case that developed periannular bleeding after mitral valve repair. While left ventricular rupture was initially suspected, the case was ultimately diagnosed as distal LCx injury based on coronary angiography findings and successfully treated with coil embolization.

### Case presentation

A 79-year-old male with a history of complete atrioventricular block treated with permanent pacemaker implantation 12 years earlier was referred to our hospital for the surgical treatment of severe mitral regurgitation. The patient’s medical history included hypertension, chronic kidney disease, and hyperuricemia. The patient had a 30-year smoking history (20 cigarettes per day) and reported drinking approximately 350 mL of beer per day.

The patient had experienced progressive exertional dyspnea for several years and had been hospitalized at a referring hospital four months earlier for worsening heart failure. Further evaluation revealed severe mitral regurgitation and the patient was referred to our institution for surgery. Paroxysmal atrial fibrillation was also diagnosed during the preoperative evaluation.

Preoperative transthoracic echocardiography showed a left ventricular ejection fraction of 63% and severe mitral regurgitation. Transesophageal echocardiography demonstrated prolapse of the P1 segment of the posterior mitral leaflets. Preoperative coronary angiography revealed no significant coronary artery stenoses. Electrocardiography exhibited a paced rhythm of 80 beats/minute.

Based on these findings, mitral valve repair, pulmonary vein isolation, and left atrial appendage closure were planned.

### Operation

The operation was performed via median sternotomy under general anesthesia. After cardiopulmonary bypass, pulmonary vein isolation was performed using a bipolar radiofrequency ablation system (AtriCure Inc., Mason, OH, USA). Left atrial appendage closure was performed using an AtriClip device (AtriCure Inc., Mason, OH, USA).

The aorta was then cross-clamped and cardiac arrest was achieved with cardioplegia. Mitral valve repair was performed for P1 prolapse. One pair of artificial chordae (CV-4 expanded polytetrafluoroethylene suture) was anchored to the anterior papillary muscle and attached to the A1 and P1 segments. Subsequently, two commissural sutures (Magic Stitch technique using 5 − 0 polypropylene sutures) were placed at the anterolateral commissure. Saline testing demonstrated satisfactory leaflet coaptation with only trivial residual regurgitation. Thereafter, eleven interrupted 3 − 0 braided polyester sutures (Ethibond Excel; Ethicon, Somerville, NJ, USA) were placed circumferentially along the mitral annulus, and a true-sized 34-mm Carpentier-Edwards Physio II annuloplasty ring (Edwards Lifesciences, Irvine, CA, USA) was implanted. Post-repair transesophageal echocardiography demonstrated trivial mitral regurgitation without evidence of mitral stenosis.

The total operative time was 5 h 1 min, and the total blood loss was 1080 mL. Marked tissue fragility was noted intraoperatively. A subepicardial hematoma was observed around the circumflex artery. However, the hematoma remained localized beneath the epicardium without evidence of active bleeding or progressive enlargement. Following protamine administration, no further expansion of the hematoma was observed, and satisfactory hemostasis was confirmed. Therefore, chest closure was performed.

### Postoperative course

Immediately after surgery, the patient’s hemodynamic status was stable with a systolic blood pressure in the 90-mmHg range under minimal vasopressor support (noradrenaline 0.01 µg/kg/min). Approximately 30 min after surgery, drainage bleeding gradually increased and reached 450 mL within the first postoperative hour. Despite this increase, hemodynamic stability was maintained with transfusion and intravenous fluid replacement. Electrocardiography demonstrated a paced rhythm without new arrhythmias or obvious ischemic changes. However, assessment of ischemic ST-T changes was limited because of the paced rhythm.

Transesophageal echocardiography demonstrated no communication between the left ventricular cavity and the pericardial space and showed no significant pericardial effusion. However, left ventricular rupture could not be completely excluded. Therefore, an intra-aortic balloon pump was inserted to reduce left ventricular wall stress and intracavitary pressure while further diagnostic evaluation was performed, and contrast-enhanced computed tomography was performed.

**Fig. 1:**
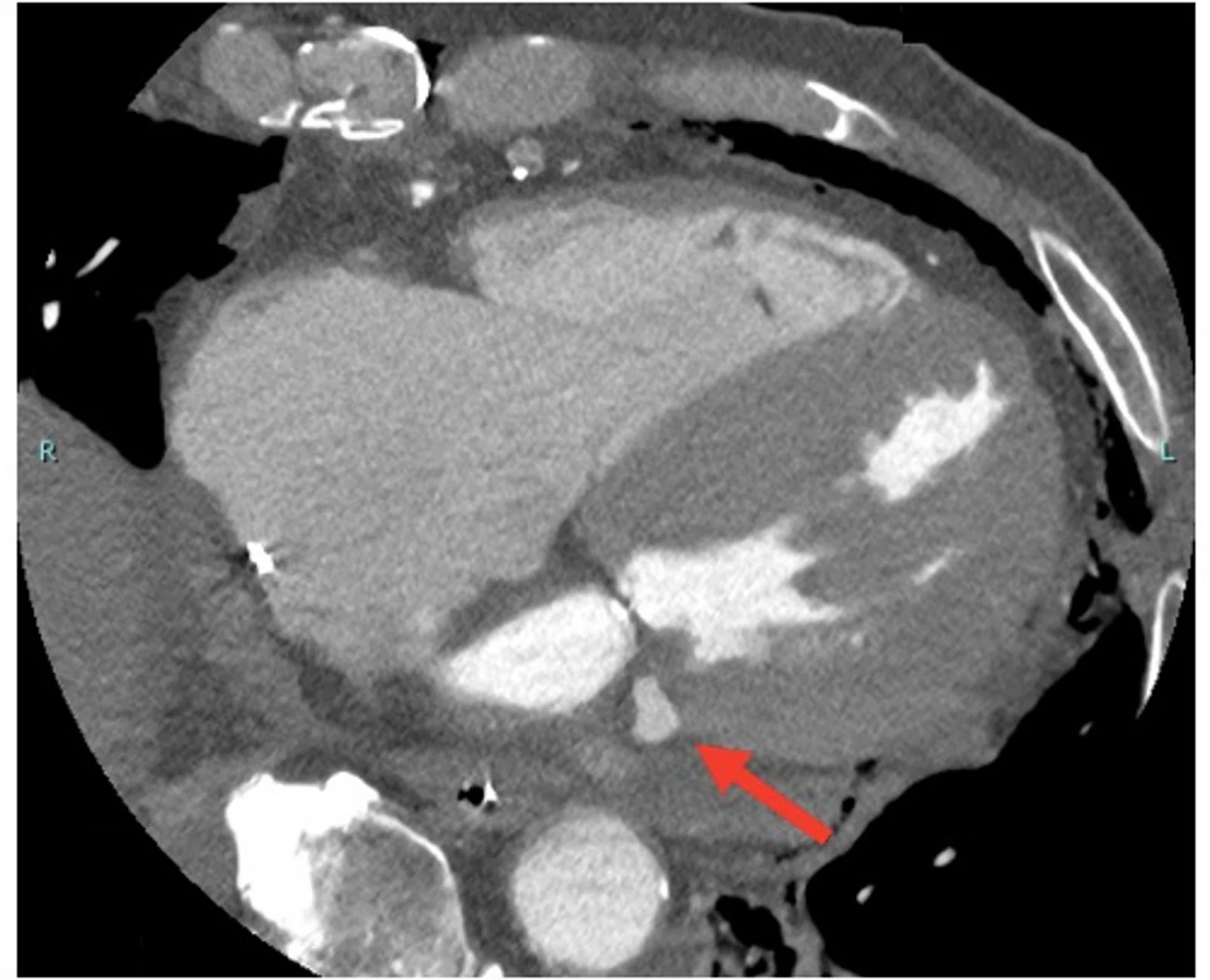
Postoperative contrast-enhanced computed tomography. Contrast-enhanced computed tomography showing contrast extravasation in the dorsal aspect of the left atrium and around the mitral annulus (arrow), suggesting active bleeding

CT revealed contrast leakage in the dorsal aspect of the left atrium and around the mitral annulus (Fig. 1), suggesting active bleeding. However, the exact source could not be identified. Because the contrast density at the leakage site was lower than that within the left ventricular cavity and the drainage volume did not decrease after IABP insertion, left ventricular rupture was considered less likely.

**Fig. 2:**
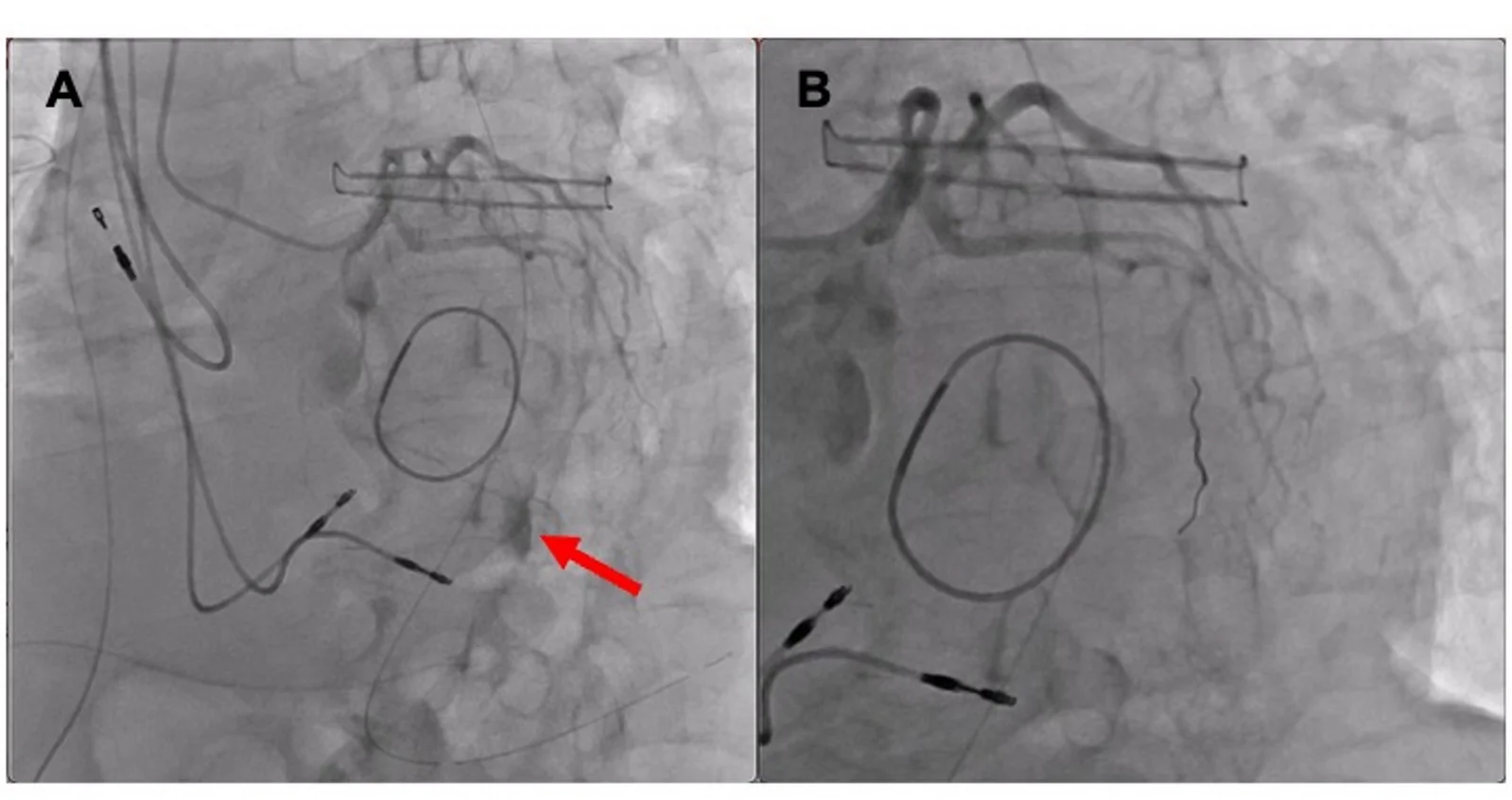
Coronary angiography and coil embolization. **A**. Coronary angiography demonstrating contrast extravasation from the distal left circumflex artery (arrow), indicating coronary artery injury. **B**. Selective coil embolization of the distal left circumflex artery showing disappearance of contrast extravasation after embolization

Coronary angiography performed for diagnostic purposes revealed contrast extravasation from the distal LCx (Fig. 2A), indicating coronary artery injury. Selective coil embolization was performed immediately (Fig. 2B), resulting in a marked reduction in drainage bleeding.

The IABP was removed on postoperative day 1. Because mild deterioration of oxygenation was observed, re-exploration was performed to remove the intrathoracic hematoma. No active bleeding was identified during re-exploration, and hematoma evacuation was required.

The patient was extubated on postoperative day 2 and transferred out of the intensive care unit on postoperative day 4. The postoperative course was uneventful, and the patient was discharged on postoperative day 18.

## Discussion

Left ventricular rupture following mitral valve surgery is one of the most catastrophic complications of cardiac surgery. Although its incidence is relatively low, ranging from 0.5% to 2%, the associated mortality remains extremely high [1, 2]. Therefore, when massive bleeding occurs after mitral valve surgery, left ventricular rupture should be considered first.

However, other causes of periannular bleeding should also be considered. The LCx runs close to the posterior mitral annulus and is vulnerable to injury during mitral valve surgery [4]. Anatomical studies have shown that the LCx may run within a few millimeters of the mitral annulus, particularly near the posterolateral commissure, making it susceptible to surgical injury.

Previous reports have described iatrogenic LCx injury associated with mitral valve surgery [4, 5]. The proposed mechanisms include direct injury from annular sutures, compression by annuloplasty rings, traction or kinking of the artery during cardiac manipulation, and external compression by a hematoma.

In the present case, massive postoperative bleeding occurred shortly after surgery, raising suspicion of left ventricular rupture or myocardial injury related to cardiac manipulation during pulmonary vein isolation. Although contrast-enhanced CT demonstrated periannular contrast leakage, the source of the bleeding could not be identified. Coronary angiography subsequently revealed extravasation from the distal LCx, confirming coronary artery injury.

The precise mechanism of LCx injury in the present case remains uncertain. Although injury related to annuloplasty sutures cannot be completely excluded, a subepicardial hematoma around the LCx was already noted intraoperatively before chest closure. Therefore, we speculate that traction or distortion of the coronary artery during cardiac manipulation for pulmonary vein isolation may have caused a minor coronary injury. Because the hematoma remained confined beneath the epicardium without evidence of active bleeding, hemostasis was considered satisfactory at that time. However, the injury may have subsequently progressed to active hemorrhage during the early postoperative period. The treatment options for LCx injuries include surgical and percutaneous coronary interventions. However, surgical hemostasis may be technically difficult in cases involving distal branches or intramyocardial segments [5]. In the present case, selective coil embolization successfully controlled the bleeding and avoided surgical re-intervention of the mitral valve.

Left ventricular rupture is suspected when massive bleeding occurs after mitral valve surgery. However, if the patient’s hemodynamic condition allows, additional diagnostic imaging should be considered before surgical re-exploration to identify the bleeding source and guide appropriate treatment strategies.

## Conclusions

We report a case of postoperative bleeding after mitral valve repair that initially suggested left ventricular rupture but was ultimately diagnosed as distal LCx injury by coronary angiography. The bleeding was successfully controlled by selective coil embolization.

When periannular bleeding occurs after mitral valve surgery, coronary artery injury should be considered. If the patient’s condition permits, additional diagnostic evaluations, including contrast-enhanced CT and coronary angiography, may help identify the bleeding source and guide appropriate treatment while avoiding unnecessary surgical reintervention.

## Data Availability

All data generated or analyzed during this study are included in this published article.

## Abbreviations

LCx: Left circumflex artery
CT: Computed tomography
IABP: Intra-aortic balloon pump
